# An Instructive CO_2_ Adsorption Model for DAC: Wave Solutions and Optimal Processes

**DOI:** 10.3390/e26110972

**Published:** 2024-11-13

**Authors:** Emily Kay-Leighton, Henning Struchtrup

**Affiliations:** Department of Mechanical Engineering, University of Victoria, Victoria, BC V8W 2Y2, Canada

**Keywords:** direct air capture, CO_2_, adsorption

## Abstract

We present and investigate a simple yet instructive model for the adsorption of CO_2_ from air in porous media as used in direct air capture (DAC) processes. Mathematical analysis and non-dimensionalization reveal that the sorbent is characterized by the sorption timescale and capacity, while the adsorption process is effectively wavelike. The systematic evaluation shows that the overall adsorption rate and the recommended charging duration depend only on the wave parameter that is found as the ratio of capacity and dimensionless air flow velocity. Specifically, smaller wave parameters yield a larger overall charging rate, while larger wave parameters reduce the work required to move air through the sorbent. Thus, optimal process conditions must compromise between a large overall adsorption rate and low work requirements.

## 1. Introduction

In 2015, at the UN Climate Change Conference (COP21), 196 parties signed the historic Paris Agreement, which set long-term goals for all nations to limit global warming to below 2 °C over pre-industrial levels [1]. More recently, however, the Intergovernmental Panel on Climate Change (IPCC) has stressed that in order to prevent the more severe impacts of climate change, the temperature increase must be limited to 1.5 °C above pre-industrial levels [2], requiring a reduction of greenhouse gas emissions by 45% from 2010 levels by 2030 and the achievement of net-zero by 2050 [3]. At COP28, in 2023, the first global stocktake was conducted to assess progress towards the Paris Agreement targets, and the key findings concluded that global emissions are not on target with the mitigation pathway consistent with the global temperature goal and that significantly more ambitious mitigation plans need to be developed and implemented to accelerate the reduction of emissions across all sectors [4].

While the path to limiting warming to 1.5 °C remains primarily driven by a reduction in emissions through behavioral changes and technological advancements and deployment, there is a growing acknowledgment of the critical role of negative emission technologies in achieving net-zero [5,6]. In particular, carbon dioxide (CO_2_) removal strategies will be essential for offsetting the remaining emissions in sectors where a complete reduction is prohibitively challenging, such as in the aviation and heavy industries [5]. Furthermore, CO_2_ removal strategies, which are still relatively new technologies, will be able to play a more significant role as the processes become more efficient and mitigation strategies begin to reach their maximum potential [6]. Thus, research, development, and the commercialization of carbon removal is of current importance so that the technology can be deployed at scale in the near term [6].

Carbon dioxide capture can take place in two forms: point source capture, which involves collecting carbon directly from the source of emission and direct air capture (DAC), whereby carbon is removed from ambient air. Generally, DAC is more energy-intensive and, therefore, more expensive than post-combustion point source capture, primarily due to the low concentration of CO_2_ in the atmosphere compared to emission sources such as flue gases [7]. Despite this, DAC remains a promising and potentially vital technology. An analysis of the methods of decarbonization of US natural gas power revealed that DAC is more applicable than post-combustion capture for power plants, which have a lower capacity or barriers to retrofitting and is the cheaper capture method for about one-third of all power plant emissions [8]. DAC also has the significant advantage of being capable of off-setting both past and present emissions and emissions from mobile sources. Additionally, since DAC plants do not need to be built at the source of emissions, they can be strategically located in areas with widely available renewable energy sources or in close proximity with CO_2_ storage or processing facilities. There is also some suggestion that DAC could be used in the development of an anthropogenic chemical carbon cycle, in which carbon emissions are captured and then recycled for use in fuels or other products [9,10].

As of 2022, there were 19 actively operating DAC plants collectively capturing approximately 10,000 tonnes of CO_2_ per year with an average cost of between USD 250–USD 600 per tonne of CO_2_ [5,11]. While these figures offer a promising start, they are well below the gigaton capacity and USD 100 per ton cost that will need to be realized in order for the technology to have a meaningful climate impact by 2050 [11].

There are a variety of methods of DAC, including collection using physical and chemical adsorption, electrochemical methods, electrodialysis, membranes, mineral carbonation, cryogenics, and photocatalytic CO_2_ conversion. Each method has technical, economic, and environmental considerations [12], however, technologies using reversible sorbents are the most widely investigated and the only method currently used in commercial plants [13].

The adsorption technologies utilize a cyclical process with two steps. First, the CO_2_ molecules in the air are collected on the surface of the sorbent material, which can be a liquid or solid. Then, once the material has reached capacity, the CO_2_ molecules are released through a regeneration process. The method of CO_2_ recovery depends on the type of sorbent material with currently utilized methods, including changes in temperature and pressure [14] and a change in moisture [15,16] for solid sorbents, and a series of chemical processes for liquid sorbents [17]. The use of liquid sorbents has been pursued and proven successful [17], and has advantages such as low cost and continuous operation; however, the energy requirements and system complexity are typically higher due to the chemical separation process and higher regeneration temperatures than solid sorbents [18,19].

Climeworks is a global leader in DAC technology. They are currently operating the world’s largest direct air capture and storage plant, which has a capacity of up to 4000 tonnes per year, and are in the process of launching a new plant with a capacity of up to 36,000 tonnes per year [20]. The Climeworks technology uses a temperature vacuum swing adsorption process. During the adsorption process, fans are used to pull air through the collectors, which contain a porous solid sorbent material. Carbon dioxide in the air reacts chemically with the sorbent and binds to the material. If there is humidity in the air drawn into the collector, co-adsorption of water molecules will also occur. Once the material reaches saturation, the collector closes, and the desorption process begins. A vacuum system drops the pressure in the collector, and the sorbent material is heated to 100 °C. At these conditions, the equilibrium capacity of the material is significantly reduced, and thus, the CO_2_, and if present, the water molecules, are released from the material and removed from the collector by the vacuum system. The outflowing gas steam is cooled in order to induce condensation of any water that is present, separating it from the CO_2_. When the desorption phase finishes, the sorbent material is cooled, and the collector reopens to the environment to begin another adsorption phase [20]. The collected concentrated CO_2_ is sequestered using a mineralization process conducted by Carbfix, a partner organization [21]. In the available research, the sorbent material used is APDES-NFC-FD, which is a chemisorbent composed of amine-functionalized nanofibrillated cellulose [22]; however, other materials have also been investigated for use in TVS DAC applications, such as amine-functionalized silica or alumina, carbonate on silica, and anionic resin [18].

Direct air capture technologies are relatively well-examined in the literature, and there exists a variety of sources detailing specific processes [23,24], analyzing the effectiveness of different sorbents materials [13,25], and reviewing the technical and economic characteristics of different methods [12,26,27,28]; however, the literature on the specific TVS process that is used by Climeworks is less comprehensive. The available work is primarily limited to research published by individuals associated with Climeworks and a handful of papers that are written by non-associated authors but are in reference to the Climeworks data. The experimental work includes validation and analysis of the TVS process using two different sorbent materials, analysis and modeling of the adsorption isotherms, study of stability of the adsorption material, and an evaluation of the co-adsorption of CO_2_ and H_2_O [29,30,31,32].

In complement to the experimental research, there is also literature on the development of mathematical models of the adsorption and desorption processes and theoretical analysis of the TVS method. Wurzbacher et al. [33] developed a comprehensive heat and mass transfer model of the desorption process and Deschamps et al. [34] used a mass and energy balance model in Aspen Adsorption software to evaluate the performance of the TVS technology at an industrial scale. Leonzio et al. [35] developed a mathematical model describing the adsorption and desorption processes, using it to carry out a comparative analysis of the energy requirements, removal capacities, and costs for several sorbent materials.

The current research on the TVS adsorption process for DAC includes limited but detailed experimental results and complex and comprehensive theoretical models; however, there exists a gap in the research to understand the process on a more general and fundamental level. Much of the existing work is specific to particular process set-ups and often, due to the proprietary nature of the technology does not provide full transparency of the input parameters and equations.

A full thermodynamic evaluation of adsorption-based DAC must account for the desorption stage of the process, which relies on thermal energy supply. However, in this contribution, we are interested only in the adsorption phase.

In the following, we present a simple and easy-to-apply adsorption model that provides insight into the general adsorption process without the need for detailed and accurate information about the specifics of a particular sorbent material. Specifically, we present an instructive model for the CO_2_ adsorption step in TVS DAC systems, where non-dimensionalization identifies a handful of key parameters for evaluation, namely, as material parameters for the characteristic timescale for adsorption τ and the dimensionless adsorption capacity ϕ; as a physical parameter, the sorbent layer thickness *L*; and as process parameters, the air flow velocity *v* and the charging duration $t_{\mathrm{ch}}$.

Mathematical analysis of the set of adsorption equations reveals traveling wave solutions, which are characterized by the wave parameter $\lambda =\frac{\phi +1}{\upsilon }$, that is the ratio of capacity parameter ϕ and dimensionless flow speed $\upsilon =\frac{Lv}{\tau }$. The same parameter λ governs the initial boundary value problem describing the charging of the sorbent. Thus, the rather general question for the best charging process reduces to two specific questions, namely, which value of λ and which charging duration $t_{\mathrm{ch}}$ to chose. While large flow speeds, that is small λ, yield faster charging, they require significantly more pump work; hence, one must consider the trade-off between the charging rate and work requirements.

The present model and its discussion provide a concise understanding of adsorption-based DAC processes, as it allows a systematic evaluation with few well-defined parameters. Specifically, the evaluation of the characteristic material properties τ, ϕ of the sorbent, together with the choices for process parameters λ and $t_{\mathrm{ch}}$ provides insight into the challenges and possibilities of these processes.

The remainder of this contribution proceeds as follows: In Section 2 we present the adsorption model, which is akin to a combustion model for reactive flow. Non-dimensionalization allows us to identify the relevant scales and corresponding dimensionless parameters. Their values are identified from available data [36]. Traveling wave solutions are found in Section 3, which identifies the wave parameter λ as the main process parameter. The charging processes are discussed in detail in Section 4 and are based on numerical solutions as well as wave solutions. The charging rate and duration and the corresponding work requirement are analyzed in detail. In Section 5, we explore the optimal system and process conditions, including an estimate of the required size of impactful adsorption-based DAC facilities. The paper ends with our conclusions.

The results presented below extend and refine preliminary work in the honours thesis of EKL [37].

## 2. Transport Equations

### 2.1. The Model

We describe the adsorption of CO_2_ from air as it is forced through a porous adsorbing material. Detailed adsorption models are widely available and discussed in the literature for DAC processes, e.g., in Refs. [28,33,34,35]. Our interest focuses on the overall behavior of DAC adsorption systems, for which the simple model presented below provides a meaningful foundation.

For model development, we first look at the flow through a duct, with a molar adsorption capacity at the walls denoted as $\beta \left(x,t\right)$$\left[\mathrm{unit}:\;\frac{\mathrm{mol}}{m^{2}}\right]$. The (temperature dependent) saturation capacity $\beta_{0}$ defines the maximum occupation of the surface, that is $\beta =\beta_{0}$ when all available sites are occupied. We only consider the adsorption at low temperature, as a strong process going mainly forward, similar to the simplified description of combustion processes [38]; see Appendix A for a discussion in the context of linear irreversible thermodynamics [39,40]. While the process is exothermic, the temperature does not noticeably change due to a large amount of air accompanying the CO_2_, to which the adsorption heat is dissipated; see [19] for a discussion of this for a chemical DAC process.

As CO_2_ carrying air is passing through the duct, some of the CO_2_ is absorbed and some is desorbed, which we express in the equation

(1) $$\frac{\partial \beta }{\partial t}=\frac{p_{{\mathrm{CO}}_{2}}}{\sqrt{2\pi M_{{\mathrm{CO}}_{2}}\bar{R}T}}\varpi \left(1-\frac{\beta }{\beta_{0}}\right)-K\beta ,$$

where the adsorption rate is a product of the following three terms: $\frac{p_{{\mathrm{CO}}_{2}}}{\sqrt{2\pi M_{{\mathrm{CO}}_{2}}\bar{R}T}}$$\left[\frac{\mathrm{mol}}{m^{2}s}\right]$ is the rate of the molecular collisions per area due to the thermal motion of particles as determined from kinetic theory of gases [41], ϖ$\left[1\right]$ is an overall probability for hitting an adsorption site with adsorption occurring, and $\left(1-\frac{\beta }{\beta_{0}}\right)$ is the probability that a site is unoccupied. Further, $p_{{\mathrm{CO}}_{2}}$ is the partial pressure of ${\mathrm{CO}}_{2}$ in air, *T* is the constant temperature of air and adsorption material, $\bar{R}$ is the universal gas constant, and $M_{{\mathrm{CO}}_{2}}$ is the molar mass of ${\mathrm{CO}}_{2}$. The desorption rate is proportional to occupation by means of the material parameter *K*, which is a function of temperature.

With the mole fraction $\chi =\frac{p_{{\mathrm{CO}}_{2}}}{p_{\mathrm{air}}}$ of CO_2_ and the ideal gas law $p_{\mathrm{air}}={\bar{\rho }}_{\mathrm{air}}\bar{R}T$, the equation assumes the form

(2) $$\frac{\partial \beta }{\partial t}={\bar{\rho }}_{\mathrm{air}}\sqrt{\frac{R_{{\mathrm{CO}}_{2}}T}{2\pi }}\varpi \left(1-\frac{\beta }{\beta_{0}}\right)\chi -K\beta$$

where $R_{{\mathrm{CO}}_{2}}=\frac{\bar{R}}{M_{{\mathrm{CO}}_{2}}}$ is the gas constant of CO_2_, and ${\bar{\rho }}_{\mathrm{air}}$ is the mole density of air, which we consider to be a constant; indeed, temperature and pressure changes are ignored throughout our discussion.

We assume that the transport of CO_2_ is due to convective flow with the air (velocity *v*) and diffusion in air (diffusion coefficient D), while molecules are adsorbed at the walls. Thus, the balance law for CO_2_ in terms of the mole fraction reads

(3) $${\bar{\rho }}_{\mathrm{air}}\left(\frac{\partial \chi }{\partial t}+v\frac{\partial \chi }{\partial x}-D\frac{\partial^{2}\chi }{\partial x^{2}}\right)=-\frac{\partial \beta }{\partial t}\frac{A_{\mathrm{duct}}}{V_{\mathrm{duct}}},$$

with the duct surface-to-volume ratio $\frac{A_{\mathrm{duct}}}{V_{\mathrm{duct}}}$ as a property of the material. For a cylindrical channel of radius *r* and length dx, we have

(4) $$\frac{A_{\mathrm{duct}}}{V_{\mathrm{duct}}}=\frac{2\pi rdx}{\pi r^{2}dx}=\frac{2}{r}$$

while for a general porous material, one typically finds the active area $a_{\mathrm{act}}$$\left[\frac{m^{2}}{\mathrm{kg}}\right]$ and the accessible volume $v_{\mathrm{acc}}$$\left[\frac{m^{3}}{\mathrm{kg}}\right]$ per mass of material, hence

(5) $$\frac{A_{\mathrm{duct}}}{V_{\mathrm{duct}}}=\frac{a_{\mathrm{act}}}{v_{\mathrm{acc}}}$$

Collecting the above results, we have the simple transport model

(6) $$\begin{array}{ccc} \frac{\partial \beta }{\partial t} & = & {\bar{\rho }}_{\mathrm{air}}\sqrt{\frac{R_{{\mathrm{CO}}_{2}}T}{2\pi }}\varpi \left(1-\frac{\beta }{\beta_{0}}\right)\chi -K\beta \end{array}$$

(7) $$\begin{array}{ccc} \frac{\partial \chi }{\partial t}+v\frac{\partial \chi }{\partial x}-D\frac{\partial^{2}\chi }{\partial x^{2}} & = & -\frac{a_{\mathrm{act}}}{v_{\mathrm{acc}}}\sqrt{\frac{R_{{\mathrm{CO}}_{2}}T}{2\pi }}\varpi \left(1-\frac{\beta }{\beta_{0}}\right)\chi -\frac{1}{{\bar{\rho }}_{\mathrm{air}}}\frac{a_{\mathrm{act}}}{v_{\mathrm{acc}}}K\beta \end{array}$$

This model is in agreement with the second law of thermodynamics as discussed in Appendix A for the non-dimensionalized equations.

### 2.2. Non-Dimensionalization

We now introduce dimensionless properties in order to simplify the notation, and to identify key parameters of the model. Specifically, we refer the lengthscale to the thickness of the adsorbent *L*, introduce a timescale τ that will be identified below, and consider the number of unoccupied sites β relative to available sites $\beta_{0}$, and the CO_2_ load relative to that of incoming air, that is, we set

(8) $$\tilde{x}=\frac{x}{L},\;\;\;\tilde{t}=\frac{t}{\tau },\;\;\;\tilde{\beta }=\frac{\beta }{\beta_{0}},\;\;\;\tilde{\chi }=\frac{\chi }{\chi_{\mathrm{atm}}}$$

to find at first

(9) $$\begin{array}{ccc} \frac{\partial \tilde{\beta }}{\partial \tilde{t}} & = & \tau \frac{{\bar{\rho }}_{\mathrm{air}}\chi_{\mathrm{atm}}}{\beta_{0}}\sqrt{\frac{R_{{\mathrm{CO}}_{2}}T}{2\pi }}\varpi \left(1-\tilde{\beta }\right)\tilde{\chi }-\tau K\tilde{\beta } \end{array}$$

(10) $$\begin{array}{ccc} \frac{\partial \tilde{\chi }}{\partial \tilde{t}}+\frac{\tau v}{L}\frac{\partial \tilde{\chi }}{\partial \tilde{x}}-\frac{\tau D}{L^{2}}\frac{\partial^{2}\tilde{\chi }}{\partial {\tilde{x}}^{2}} & = & -\tau \frac{a_{\mathrm{act}}}{v_{\mathrm{acc}}}\sqrt{\frac{R_{{\mathrm{CO}}_{2}}T}{2\pi }}\varpi \left(1-\tilde{\beta }\right)\tilde{\chi }+\frac{\tau }{{\bar{\rho }}_{\mathrm{air}}}\frac{a_{\mathrm{act}}}{v_{\mathrm{acc}}}\frac{\beta_{0}}{\chi_{\mathrm{atm}}}K\tilde{\beta } \end{array}$$

To proceed, we chose the timescale as

(11) $$\tau =\frac{\sqrt{2\pi }\beta_{0}}{\sqrt{R_{{\mathrm{CO}}_{2}}T}{\bar{\rho }}_{\mathrm{air}}\chi_{\mathrm{atm}}\varpi }$$

and introduce the capacity parameter

(12) $$\phi =\frac{a_{\mathrm{act}}}{v_{\mathrm{acc}}}\frac{\beta_{0}}{{\bar{\rho }}_{\mathrm{air}}\chi_{\mathrm{atm}}}.$$

as well as the desorption parameter

(13) $$k=\tau K=\frac{\sqrt{2\pi }\beta_{0}K}{\sqrt{R_{{\mathrm{CO}}_{2}}T}{\bar{\rho }}_{\mathrm{air}}\chi_{\mathrm{atm}}\varpi }$$

The definition of τ gives the timescale for adsorption from CO_2_-carrying air (where $\tilde{\chi }=1$). The capacity parameter ϕ is the ratio of the total available adsorption sites $a_{\mathrm{act}}\beta_{0}$ [unit: $\frac{m^{2}}{\mathrm{kg}}\frac{\mathrm{mol}}{m^{2}}=\frac{\mathrm{mol}}{\mathrm{kg}}$] and the number of CO_2_ molecules in air-filled pores $v_{\mathrm{acc}}{\bar{\rho }}_{\mathrm{air}}\chi_{\mathrm{atm}}$ [unit: $\frac{m^{3}}{\mathrm{kg}}\frac{\mathrm{mol}}{m^{3}}1=\frac{\mathrm{mol}}{\mathrm{kg}}$], both considered per mass of adsorbent.

Moreover, dimensionless velocity and the diffusion coefficient are

(14) $$\upsilon =\frac{\tau v}{L},\;\;\;D=\frac{\tau D}{L^{2}}.$$

To ease notation, we will in the following not write the tildes, that is, we write the dimensionless transport equations as

(15) $$\begin{array}{ccc} \frac{\partial \beta }{\partial t} & = & \left(1-\beta \right)\chi -k\beta \end{array}$$

(16) $$\begin{array}{ccc} \frac{\partial \chi }{\partial t}+\upsilon \frac{\partial \chi }{\partial x}-D\frac{\partial^{2}\chi }{\partial x^{2}} & = & -\phi \left[\left(1-\beta \right)\chi -k\beta \right] \end{array}$$

Specifically, β is the relative amount of local adsorption, with β=0 if all sites are free, and β=1 when all sites are occupied; χ denotes the CO_2_ load of the airflow, rescaled such that χ=1 for the incoming flow and χ=0 when all CO_2_ is removed. The parameters of the system are the material dependent coefficients τ, ϕ, k, and *D*, which are considered to be constant, and the airflow velocity υ is the only process parameter.

We point out that this model ignores the adsorption of water molecules that compete with CO_2_ and thus reduce the overall adsorption of CO_2_. Thus, the estimates below must be considered as a best case.

### 2.3. Typical Parameter Values

While we are interested in the principal behavior of the process rather than a detailed evaluation of realistic systems, it is worthwhile to have a look at the available data. Due to the proprietary nature of materials in development for industrial applications, detailed material data to relate to and fit our model is scarce. Thus, while a comprehensive examination of data for different materials would be interesting and desirable, we consider only one specific material presented in the patent by Climeworks [36], from which we extracted the values listed in Table 1.

For the fitting, we assumed that at the low temperatures during adsorption the desorption rate *K* is sufficiently small, so that the desorption processes can be ignored, that is, we set k=0. This implies that the equilibrium site density $\beta_{eq}$ equals the adsorption site density $\beta_{0}$; all subsequent results rely on this simplifying assumption. See Appendix A for an additional discussion of the influence of the desorption parameter (i.e., the dimensionless equilibrium constant).

The timescale τ (11) is inversely dependent on the probability parameter ϖ, which, at best, is obtained from a comparison to the experimental results based on the solutions of the model. For the data from [36], our fitting resulted in the values

$$\begin{array}{cccc} llll\tau =\frac{\sqrt{2\pi }\beta_{0}}{\sqrt{R_{{\mathrm{CO}}_{2}}T}{\bar{\rho }}_{\mathrm{air}}\chi_{\mathrm{atm}}\varpi } & =1.14\times 10^{4}\;s=2.8\;h & \; & \mathrm{characteristic}\;\mathrm{timescale}\\ \varpi & =2.5\times 10^{-10} & \; & \mathrm{probability}\;\mathrm{parameter} \end{array}$$

For this data, at 2.8 h, the characteristic timescale is quite large. Obviously, materials with shorter characteristic times are advantageous for technical processes since the turnover time will be proportional to τ. The parameter ϖ in our simplified model is the probability for adsorption when a CO_2_ molecule hits an adsorption site. For the data given, a molecule must hit the surface about $4\times 10^{9}$ times before it is adsorbed.

For this data, the dimensionless parameters appearing in the model assume the values

$$\begin{array}{cccc} \upsilon =\frac{\tau v}{L} & =7620 & \; & \mathrm{dimensionless}\;\mathrm{air}\;\mathrm{velocity}\\ D=\frac{\tau D}{L^{2}} & =114 & \; & \mathrm{dimensionless}\;\mathrm{diffusion}\;\mathrm{coefficient}\\ \phi =\frac{a_{\mathrm{act}}}{v_{\mathrm{acc}}}\frac{\beta_{0}}{{\bar{\rho }}_{\mathrm{air}}\chi_{\mathrm{atm}}} & =43770 & \; & \mathrm{capacity}\;\mathrm{parameter}\\ k=\tau K & \ll 1 & \; & \mathrm{desorption}\;\mathrm{parameter}\;(\mathrm{ignored}) \end{array}$$

With velocity and diffusivity quite different in size, such that $\frac{D}{\upsilon }=\frac{D}{vL}\ll 1$ processes are dominated by convection while diffusion can be ignored, we have set D=0 from now on.

### 2.4. CO_2_ Conservation

The conservation law for CO_2_ molecules is obtained from Equations (15) and (16) by elimination of the adsorption rates as

(17) $$\frac{\partial \chi +\phi \beta }{\partial t}+\frac{\partial \upsilon \chi }{\partial x}=0$$

We consider a domain of length L=1 and a process of duration $t_{e}$.

Integration about the domain length yields

(18) $$\frac{d}{dt}\int_{0}^{1}\left(\chi \left(x,t\right)+\phi \beta \left(x,t\right)\right)dx+\upsilon \left[\chi \left(1,t\right)-\chi \left(0,t\right)\right]=0$$

and subsequent integration over the process duration $t_{e}$ yields

(19) $$\upsilon \int_{0}^{t_{e}}\left[\chi \left(0,t\right)-\chi \left(1,t\right)\right]dt=\phi \int_{0}^{1}\left[\beta \left(x,t_{e}\right)-\beta \left(x,0\right)\right]dx+\int_{0}^{1}\left[\chi \left(x,t_{e}\right)-\chi \left(x,0\right)\right]dx.$$

The expression on the left of this equation is the difference between the overall inflow (at x=0) and outflow (at x=1), while the expression on the right is the overall amount of the CO_2_ adsorbed (ϕβ) and present as gas (χ) at $t_{e}$.

## 3. Wave Solution

When diffusion can be ignored, the transport model yields traveling wave solutions, which we explore in this section. As will be seen, the analytical travelling wave solutions describe the processes very well, apart from the initial stage of the process. With that, they are very helpful for the better understanding of process behavior.

For the computations, it is advantageous to use the relative number of unoccupied adsorption sites, defined as

(20) $$\hat{\beta }=1-\beta$$

with $\hat{\beta }=1$ when all sites are free, and $\hat{\beta }=0$ when all sites are occupied. With this, and vanishing diffusivity *D*, the transport Equations (15) and (16) reduce to

(21) $$\begin{array}{ccc} \frac{\partial \hat{\beta }}{\partial t} & = & -\hat{\beta }\chi \end{array}$$

(22) $$\begin{array}{ccc} \frac{\partial \chi }{\partial t}+\upsilon \frac{\partial \chi }{\partial x} & = & -\phi \hat{\beta }\chi \end{array}$$

Note that transport is linear, but decay is non-linear.

### 3.1. Travelling Waves

We assume that both functions are traveling waves with an unknown wave speed αυ proportional to the flow velocity υ, so that traveling wave solutions are of the form

(23) $$\begin{array}{ccc} \hat{\beta }\left(x,t\right) & = & \hat{\beta }\left(x-\alpha \upsilon t\right)=\hat{\beta }\left(\xi \right) \end{array}$$

(24) $$\begin{array}{ccc} \chi \left(x,t\right) & = & \chi \left(x-\alpha \upsilon t\right)=\chi \left(\xi \right) \end{array}$$

where ξ=x−αυt is the variable for the traveling wave. The velocity factor α will be determined as we proceed.

We denote differentiation with respect to the wave variable ξ by prime, which gives the transport equations as

(25) $$\begin{array}{ccc} \alpha \upsilon {\hat{\beta }}^{\prime } & = & \hat{\beta }\chi \end{array}$$

(26) $$\begin{array}{ccc} -\alpha \upsilon \chi^{\prime }+\upsilon \chi^{\prime } & = & -\phi \hat{\beta }\chi \end{array}$$

which combine to

(27) $${\hat{\beta }}^{\prime }=-\frac{\left(1-\alpha \right)}{\phi \alpha }\chi^{\prime }.$$

Integration yields

(28) $$\hat{\beta }=-\frac{\left(1-\alpha \right)}{\phi \alpha }\chi +C$$

with the constant of integration *C*.

With this, the second equation becomes an ODE for χ that can be written in the compact form

(29) $$\chi^{\prime }=\frac{\chi^{2}}{\alpha \upsilon }-\lambda \chi ,$$

where the wave parameter λ—which will play an important role in the following—is introduced as

(30) $$\lambda =\frac{\phi C}{\left(1-\alpha \right)\upsilon }\;.$$

Integration yields, with the constant of integration Γ,

(31) $$\left(\frac{1}{\chi }-\frac{1}{\lambda \alpha \upsilon }\right)=\Gamma \exp\left[\lambda \xi \right],$$

hence the general wave solution reads

(32) $$\chi =\frac{\lambda \alpha v}{\Gamma \lambda \alpha \upsilon \exp\left[\lambda \xi \right]+1}.$$

### 3.2. Determination of Constants of Integration

The constants α, λ (or *C*), and Γ are identified as follows:

With υ>0, the wave travels from left to right connecting states of CO_2_-carrying air, with $\chi \left(\xi \to -\infty \right)=1$, and fully depleted air, with $\chi \left(\xi \to \infty \right)=0$. The solution (32) yields the second condition for all λ>0, while the first condition demands that the wave parameter is the inverse wave speed

(33) $$\lim_{\xi \to -\infty }\frac{\lambda \alpha \upsilon }{\lambda \alpha \upsilon \Gamma \exp\left[\lambda \xi \right]+1}=\lambda \alpha \upsilon \overset{!!}{=}1\;\;\;\Rightarrow \;\;\;\lambda =\frac{1}{\alpha \upsilon }$$

With this, the solution for the relative CO_2_ load reads

(34) $$\chi \left(\xi \right)=\frac{1}{1+\exp\left[\frac{1}{\alpha \upsilon }\left(\xi -\xi_{0}\right)\right]}$$

where the offset $\xi_{0}$ is defined through $\lambda \alpha v\Gamma =\exp\left[-\lambda \xi_{0}\right]$; note that negative values of Γ give divergent results, hence are not physical.

We are now left with one last problem, which is the question of the value of the velocity factor α. For this, we consider the solution for the site occupation $\hat{\beta }=1-\beta$, which from (28), (30), (33), and (34) becomes

(35) $$\hat{\beta }=\frac{1-\alpha }{\phi \alpha }\left[\frac{1}{1+\exp\left[-\lambda \left(\xi -\xi_{0}\right)\right]}\right]$$

In the limit ξ→∞, we have all unoccupied sites, hence $\hat{\beta }=1$ and

(36) $$1=\hat{\beta }\left(\xi \to \infty \right)=\lim_{\xi \to -\infty }\left[\frac{1-\alpha }{\phi \alpha }\left[\frac{1}{1+\exp\left[-\lambda \left(\xi -\xi_{0}\right)\right]}\right]\right]=\frac{1-\alpha }{\phi \alpha }$$

which gives the velocity factor and wave parameter as

(37) $$\alpha =\frac{1}{\phi +1},\;\;\;\lambda =\frac{\phi +1}{\upsilon }$$

Note that with this

(38) $$\hat{\beta }=\frac{1}{1+\exp\left[-\frac{\phi +1}{\upsilon }\left(\xi -\xi_{0}\right)\right]}\;\;\;⟹\;\;\;\beta =1-\hat{\beta }=\frac{1}{1+\exp\left[\lambda \left(\xi -\xi_{0}\right)\right]}=\chi .$$

that is, for the wave solution $\beta \left(x,t\right)=\chi \left(x,t\right)$. This simple relation results from ignoring the desorption rate. For the non-zero desorption rate k wave parameter, the width of the wave and equilibrium conditions are affected by k, while the general behavior is the same as for the simpler case k=0.

### 3.3. Wave Structure

Combining the results from above, we find the traveling wave solution as

(39) $$\chi \left(\xi \right)=\beta \left(\xi \right)=\frac{1}{1+\exp\left[\lambda \left(\xi -\xi_{0}\right)\right]}=\frac{1}{1+\exp\left[\lambda \left(x-\xi_{0}\right)-t\right]};$$

that is, χ and β agree for the waves. Notably, the structure of the wave is determined only through the value of the wave parameter $\lambda =\frac{\phi +1}{\upsilon }$.

**Figure 1:**
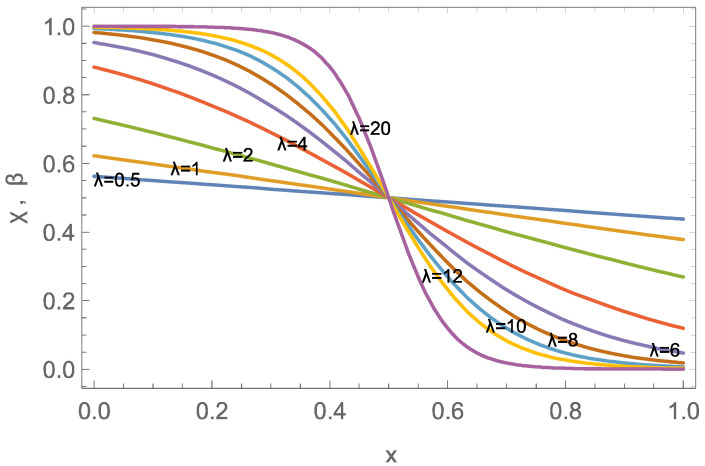
Wave solution centered at x=0.5 for wave parameters $\lambda =\frac{\phi +1}{\upsilon }$ in $\left[0.5,\;20\right]$.

Figure 1 shows the structure of the wave for a variety of wave parameters $\lambda =\frac{\phi +1}{\upsilon }\in \left[0.5,\;20\right]$, centered in the accessible domain

Wave-like transport will be observed when the domain width *L* is larger than the effective width of the wave, which we approximate as the distance between points $x_{0}$, $x_{1}$ where the values of β are $\beta_{th}$ and $1-\beta_{th}$, respectively, so that

(40) $$\Delta x=x_{0}-x_{1}=\frac{1}{\lambda }\left[\ln\frac{1-\beta_{th}}{\beta_{th}}-\ln\frac{\beta_{th}}{1-\beta_{th}}\right]=\frac{2}{\lambda }\ln\frac{1-\beta_{th}}{\beta_{th}}$$

We note that the width is linear in the inverse wave parameter $\frac{1}{\lambda }=\frac{\upsilon }{\phi +1}$, hence small velocities υ or large capacities ϕ give more compact waves, while the waves are wide for fast flows or low capacity.

Setting the threshold value to $\beta_{th}=0.05$ gives an approximate dimensionless width $\Delta x=\frac{6}{\lambda }$. Hence, full wave structures will be observed in the domain (L=1) for

(41) $$\Delta x≲1\;\;\;⟹\;\;\;\;\;\lambda =\frac{\phi +1}{\upsilon }≳6.$$

For the example data, $\lambda =\frac{\phi +1}{\upsilon }=5.75$, hence wave-dominated transport is expected.

## 4. Charging Processes

### 4.1. Initial Boundary Value Problem

The charging process of the sorbent is the solution of our dimensionless model

(42) $$\begin{array}{ccc} \frac{\partial \beta }{\partial t} & = & \left(1-\beta \right)\chi \end{array}$$

(43) $$\begin{array}{ccc} \frac{\partial \chi }{\partial t}+\upsilon \frac{\partial \chi }{\partial x} & = & -\phi \left(1-\beta \right)\chi \end{array}$$

for the case that initially no CO_2_ is in the domain, and all adsorption sites are free, that is

(44) $$\mathrm{initial}\;\mathrm{conditions}\text{:}\;\;\;\chi \left(x,t=0\right)=\beta \left(x,t=0\right)=0$$

Only one boundary condition is required, which is the (dimensionless) mole fraction of incoming air at x=0:

(45) $$\mathrm{boundary}\;\mathrm{condition}\text{:}\;\;\;\chi \left(x=0,t\right)=1.$$

Airflow velocity υ is the only process parameter, while the capacity ϕ is a material parameter; note that the value of $\upsilon =\frac{\tau v}{L}$ depends on the time- and length-scale, which are properties of the material.

**Figure 2:**
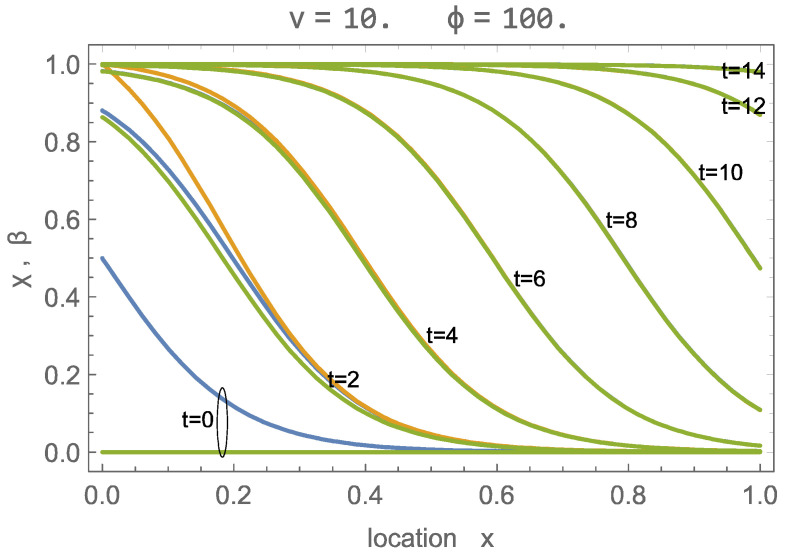
Solution of adsorption process with υ=10, ϕ=100, that is $\lambda =\frac{\phi +1}{\upsilon }=10.1$, for times t=0,2,4,…,14; green: $\beta \left(x,t\right)$; orange: $\chi \left(x,t\right)$; blue: wave solution for χ,β.

The numerical solution to this problem is straightforward: we used the NDSolve function of Wolfram Mathematica. Figure 2 shows, for υ=10, ϕ=100, or $\lambda =\frac{\phi +1}{\upsilon }=10.1$, and the curves for $\chi \left(x,t\right)$ (orange) and $\beta \left(x,t\right)$ (green) over the domain at various times, as well as the wave solution (blue), where the wave shift $\xi_{0}$ was adjusted to provide agreement at larger times.

Figure 2 shows initial differences between χ and β, as well as their wave solutions. However, as the process proceeds, the numerical solution develops into the traveling wave solution, with all three curves agreeing well for times t≥4. The flow velocity υ is relatively slow so that all CO_2_ is adsorbed soon after entering. At time τ=14, the adsorption sites are almost fully occupied.

A further evaluation shows that the shift $\xi_{0}$ in the wave solution is independent of the parameter values. Specifically, we found that a shift of zero gives excellent long-time agreement with numerical solutions for all values at the parameters. With this, at the beginning of the process, the wave structure is centered at the inlet (x=0),

(46) $$\mathrm{wave}\;\mathrm{solution}\text{:}\;\chi \left(x,t\right)=\beta \left(x,t\right)=\frac{1}{1+\exp\left[\lambda x-t\right]}.$$

**Figure 3:**
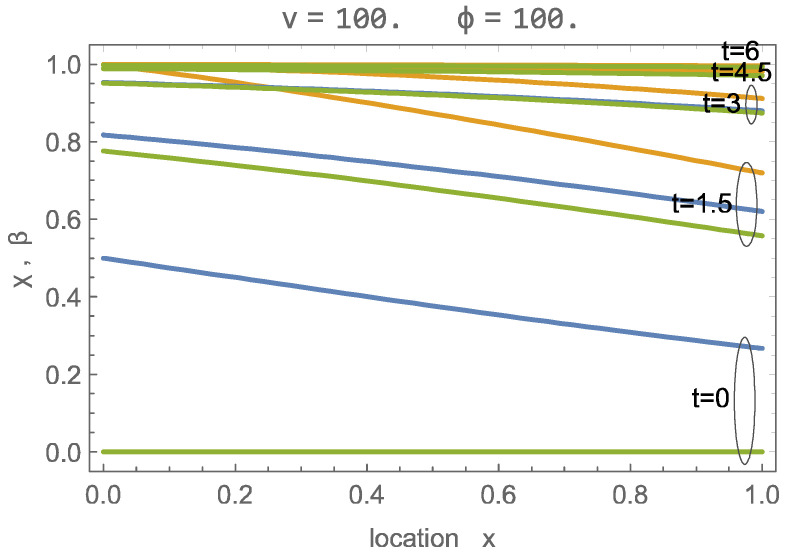
Solution of adsorption process with $\lambda =\frac{\phi +1}{\upsilon }=1.01$ for υ=100, ϕ=100, for times t=0,1.5,3,4.5,6; green: $\beta \left(x,t\right)$; orange: $\chi \left(x,t\right)$; blue: wave solution for χ,β.

Figure 3 shows the numerical and wave solutions for a fast charging process, at $\lambda =\frac{\phi +1}{\upsilon }=1$, for which the signal width is considerably wider than the domain. Due to the large velocity, an abundance of CO_2_ is provided so that adsorption occurs at all locations simultaneously, and all sites are filled at about t=4.5. While the wave solution deviates in the early stages, from t=4 forward, the agreement is quite good.

For the results above, we have given the values of velocity υ and capacity ϕ separately. A notable outcome of our evaluation is that the solution behavior—numerical or wave solution—is determined only by the value of the wave parameter $\lambda =\frac{\phi +1}{\upsilon }$, which is inversely proportional to the width of fully developed waves. Thus, in the following, we will not refer to the individual values for υ, ϕ but only to the values of the wave parameter λ, which is one of two process parameters to describe the charging of a sorbent; the other will be identified as the charging duration $t_{\mathrm{ch}}$.

### 4.2. Charging Duration

We proceed with the discussion of the required process duration to fill the adsorption sites to a certain level.

The CO_2_ accumulation, which is the relative amount of CO_2_ collected at time *t*, is the space average (with L=1 in the dimensionless formulation),

(47) $$\bar{\beta }\left(t\right)=\int_{0}^{1}\beta \left(x,t\right)dx.$$

For the wave solution, the corresponding integral

(48) $$\bar{\beta }\left(t\right)=\int_{0}^{1}\frac{1}{1+\exp\left[\frac{\phi +1}{\upsilon }x-t\right]}dx$$

can be solved to give the CO_2_ accumulation as an explicit function of time *t* and wave parameter $\lambda =\frac{\phi +1}{\upsilon }$,

(49) $$\bar{\beta }\left(t\right)=1+\frac{1}{\lambda }\ln\left[\frac{1+\exp\left[-t\right]}{1+\exp\left[\lambda -t\right]}\right].$$

As can be seen already in Figure 2 and Figure 3, the wave solution does not properly predict the early stages of charging, as reflected in the non-zero values of $\bar{\beta }\left(t=0\right)$, which can be as large as 0.5 for λ→0.

**Figure 4:**
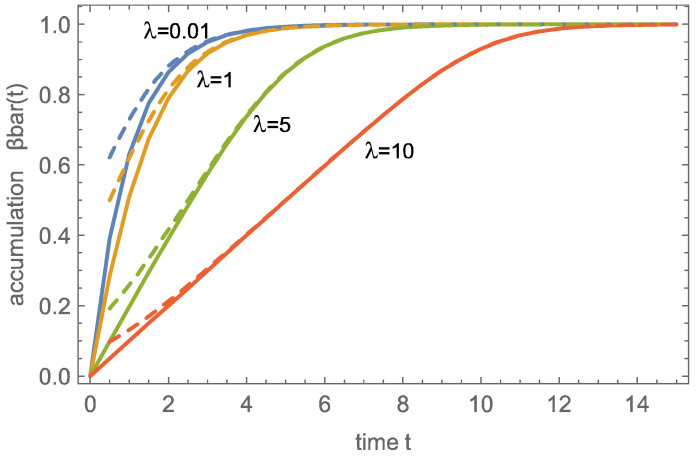
Accumulation $\bar{\beta }\left(t\right)$ over time for $\lambda =\frac{\phi +1}{v}=0.01,\;1,\;5,\;10;$ continuous: numerical solution; dashed: wave solution.

Figure 4 shows the accumulation over time for four values of λ, comparing the numerical result (continuous) with the wave solution (dashed), again with good agreement for larger times as well as larger accumulations $\bar{\beta }$. For larger λ, the accumulation results from the fully developed wave traveling through the domain, which leads to the linear charging behavior that is clearly visible for λ=5,10.

An important question for applications is how long the process should run to make the best use of the sorbent material. We define the charging duration $t_{\mathrm{ch}}$ as the time required to reach a desired accumulation value ${\bar{\beta }}_{\mathrm{ch}}$.

The wave solution allows a quick evaluation of the charging time, which is found from the inversion of (49) as

(50) $$t_{\mathrm{ch}}=\ln\frac{\exp\left[\lambda {\bar{\beta }}_{\mathrm{ch}}\right]-1}{1-\exp\left[-\lambda \left(1-{\bar{\beta }}_{\mathrm{ch}}\right)\right]}.$$

**Figure 5:**
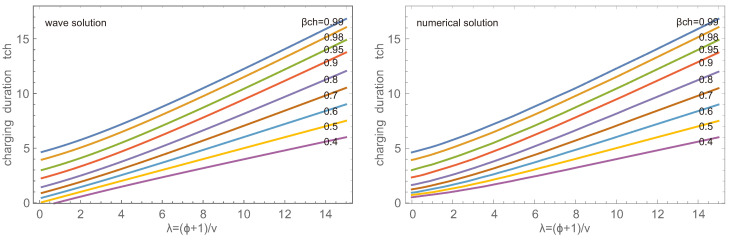
Charging duration $t_{\mathrm{ch}}$ in dependence of $\lambda =\frac{\phi +1}{\upsilon }$ for accumulations ${\bar{\beta }}_{\mathrm{ch}}=0.4,\ldots ,0.99$ for wave (left) and numerical (right) solutions.

Accordingly, the charging duration increases with $\lambda =\frac{\phi +1}{\upsilon }$ as well as with the desired value ${\bar{\beta }}_{\mathrm{ch}}$, as shown in Figure 5 for the wave and numerical solution. Note that for the wave solution ${\bar{\beta }}_{\mathrm{ch}}\left(0\right)\neq 0$ since the wave solution does not match the proper initial condition $\beta \left(x,0\right)=0$. Thus the durations for small λ and small ${\bar{\beta }}_{\mathrm{ch}}$ differ between wave and numerical solutions, while they agree well for larger λ and ${\bar{\beta }}_{\mathrm{ch}}$. The plot gives a clear indication that charging to a large accumulation takes particularly long. Accordingly, in applications, one will not aim to charge to, say, 98%, but rather to a lower value and run the charge-desorption cycle more often.

### 4.3. Overall Charging Rate

For the best use of the sorbent in applications, one will be interested in large accumulations ${\bar{\beta }}_{\mathrm{ch}}$ and short charging duration $t_{\mathrm{ch}}$, that is, the large overall charging rates

(51) $${\dot{\beta }}_{\mathrm{ch}}=\frac{{\bar{\beta }}_{\mathrm{ch}}\left(t_{\mathrm{ch}}\right)}{t_{\mathrm{ch}}}.$$

**Figure 6:**
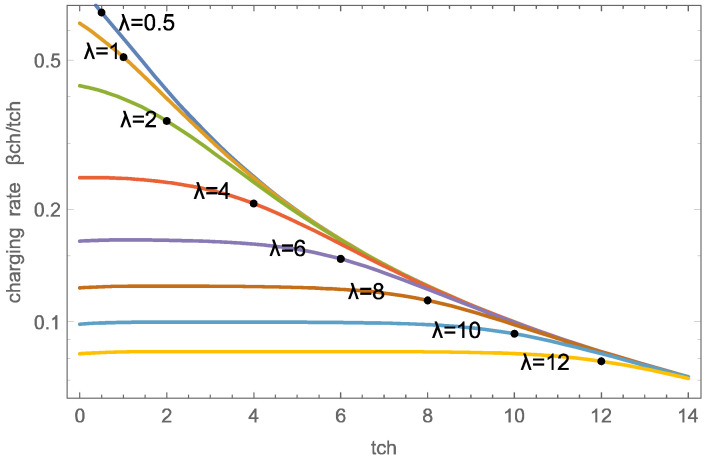
Charging rates $\dot{\beta }={\bar{\beta }}_{\mathrm{ch}}$/$t_{\mathrm{ch}}$ as functions of charge duration $t_{\mathrm{ch}}$ for several $\lambda =\frac{\phi +1}{\upsilon }$ (numerical solution). The dots indicate the values at $t_{\mathrm{ch}}=\lambda$. Note the logarithmic scale.

Figure 6 shows the charging rate ${\dot{\beta }}_{\mathrm{ch}}$ as a function of charging time $t_{\mathrm{ch}}$ for the wave parameters $\lambda \in \left[0.5,\;12\right]$ in a logarithmic scale. For larger durations, the overall rate ${\dot{\beta }}_{\mathrm{ch}}$ decreases, hence it is advantageous to not fully charge the sorbent, but rather terminate charging earlier, and charge more often.

For smaller λ, that is large velocities, the wave width is larger than the domain, hence, as depicted in Figure 3, charging is distributed through the domain, with short charging durations yielding large rates.

For λ≥4, the charging rates are initially flat due to the wave-dominated charging process depicted in Figure 2. The exiting wave is centered at the domain exit (x=1) for t=λ; hence the charging rate decreases around that time.

Not surprisingly, the figure indicates significantly higher charging rates for smaller $\lambda =\frac{\phi +1}{\upsilon }$, that is, a faster airflow. As will be discussed below, large velocities require considerably larger pump work and, thus, are not favorable for efficiency processes.

The dots in the figure indicate $t_{\mathrm{ch}}=\lambda$, which appears to be a meaningful charging duration for all cases. Indeed, there would be little gain in charging with λ=0.5 until $t_{\mathrm{ch}}=2$, which gives almost the same overall rate as charging with λ=1 until $t_{\mathrm{ch}}=2$, but requires significantly more pump work to maintain the large flow velocity υ.

Table 2 shows accumulations ${\bar{\beta }}_{\mathrm{ch}}$ and overall rates ${\dot{\beta }}_{\mathrm{ch}}$ for the case $t_{\mathrm{ch}}=\lambda$ for the numerical and wave solutions, which agree well for λ≥4.

Notably, in particular for small λ, it is advantageous to not charge the sorbent to large accumulation, but rather to a lower accumulation (e.g., ${\bar{\beta }}_{\mathrm{ch}}=0.509$ for λ=1) and more often.

### 4.4. Breakthrough Curve

We note that the accumulation $\bar{\beta }\left(t\right)$ is not accessible from direct measurements. Nevertheless, it can be determined from the breakthrough curve, i.e., the ratio of CO_2_ mole fractions in the exiting and entering airflows [35], which both can be measured; in our dimensionless variables the breakthrough curve is given by

(52) $$\frac{\chi \left(1,t\right)}{\chi \left(0,t\right)}=\chi \left(1,t\right),$$

since $\chi \left(0,t\right)=1$, which is due to the definition of χ and the boundary condition (45). To proceed, we recall the space integrated conservation of ${\mathrm{CO}}_{2}$ (18),

(53) $$\frac{d}{dt}\int_{0}^{1}\left(\chi \left(x,t\right)+\phi \beta \left(x,t\right)\right)dx+\upsilon \left[\chi \left(1,t\right)-\chi \left(0,t\right)\right]=0$$

For any meaningful adsorption material, the capacity is large, ϕ≫1, and we have seen that $\chi \left(x,t\right)\to \beta \left(x,t\right)$ for larger *t*. Hence, with $\chi \left(0,t\right)=1$ and the definition (47) of the accumulation, we can approximate the conservation law as

(54) $$\frac{d\bar{\beta }\left(t\right)}{dt}=\frac{\upsilon }{\phi +1}\left[1-\chi \left(1,t\right)\right]=\frac{1}{\lambda }\left[1-\chi \left(1,t\right)\right].$$

Integration over the duration of the process, with $\bar{\beta }\left(t=0\right)=0$, yields the accumulation at time *t* as

(55) $$\bar{\beta }\left(t\right)=\frac{1}{\lambda }\left[t-\chi_{\mathrm{out}}\left(t\right)\right],$$

where

(56) $$\chi_{\mathrm{out}}\left(t\right)=\int_{0}^{t}\chi \left(1,t^{\prime }\right)dt^{\prime }.$$

Hence, the accumulation $\bar{\beta }\left(t\right)$ can be determined from the time integration of the breakthrough curve and the wave parameter λ, which must be determined from other measurements.

### 4.5. Excess Air

The capacity parameter ϕ depends only on the material; hence, it is unaffected by the process parameters. From the previous discussion, it is evident that smaller values of the wave parameter $\lambda =\frac{\phi +1}{\upsilon }$, that is, larger flow speeds υ, are advantageous since they yield faster charging; hence, a better turn-around of charge–discharge processes and a better use of the sorbent. Next, we explore the relation between the faster flow and the total amount of air required, which, indeed, grows with increasing speed.

When $\chi_{\mathrm{out}}\neq 0$, some CO_2_ leaves the domain, which implies that excess airflow is required to provide the unused CO_2_. Since work is required to force air through the porous sorbent, a larger air requirement is undesirable. To quantify excess air, we consider the amount of CO_2_ collected relative to the CO_2_ inflow with air, that is, the relative air usage over the charging duration $t_{\mathrm{ch}}$, defined as the ratio of accumulated CO_2_ to the inflow:

(57) $$\Pi =\frac{\upsilon \left(\chi_{\mathrm{in}}\left(t_{\mathrm{ch}}\right)-\chi_{\mathrm{out}}\left(t_{\mathrm{ch}}\right)\right)}{\upsilon \chi_{\mathrm{in}}\left(t_{\mathrm{ch}}\right)}=1-\frac{\chi_{\mathrm{out}}\left(t_{\mathrm{ch}}\right)}{\chi_{\mathrm{in}}\left(t_{\mathrm{ch}}\right)}$$

where

(58) $$\chi_{\mathrm{in}}\left(t\right)=\int_{0}^{t}\chi \left(0,t^{\prime }\right)dt^{\prime }=\int_{0}^{t}dt^{\prime }=t.$$

is the time integral over the mole fraction at the inlet.

With (55) and (51), the air usage is directly related to the charging rate and the wave parameter,

(59) $$\Pi =\lambda \frac{\bar{\beta }\left(t_{\mathrm{ch}}\right)}{t_{\mathrm{ch}}}=\lambda {\dot{\beta }}_{\mathrm{ch}}.$$

With the suggested charging duration $t_{\mathrm{ch}}=\lambda$, the air usage equals dimensionless accumulation, $\Pi =\bar{\beta }\left(t_{\mathrm{ch}}\right)$. Depending on the value of λ, air usage can be as small as 33% for λ=0.5, that is, for large velocities υ.

### 4.6. Pump Work

Low air usage Π implies that considerable pump work must be used to push (or pull) excess air through the sorbent since not all CO_2_ in the air can be collected. We proceed with determining the pump work required per mole of captured gas.

The pumping power to push a gas (i.e., air) through a porous medium of cross section *A* is given by the product of volume flow $\dot{V}=Av$ and pressure difference across the thickness *L* of the material:

(60) $$\dot{W}=\dot{V}\Delta p=Av\Delta p.$$

The pressure difference is given by the Ergun equation [42]

(61) $$\Delta p=\left(\frac{\mu_{\mathrm{air}}}{k}v_{s}+\frac{\rho_{\mathrm{air}}}{k_{1}}v_{s}^{2}\right)L,$$

where $\rho_{\mathrm{air}}$ and $\mu_{\mathrm{air}}$ are the mass density and viscosity of air, respectively,

(62) $$v_{s}=\varepsilon v$$

The superficial velocity with the void fraction ε, and permeability *k* and inertial permeability $k_{1}$ are given as

(63) $$k=\frac{d_{p}^{2}\varepsilon^{3}}{150{\left(1-\varepsilon \right)}^{2}},\;\;\;k_{1}=\frac{d_{p}\varepsilon^{3}}{1.75\left(1-\varepsilon \right)}$$

with $d_{p}$ being the particle diameter of the sorbent.

The total work for pumping air through for the charging duration $\Delta t=\tau t_{\mathrm{ch}}$ is, with $v=\frac{L}{\tau }\upsilon$,

(64) $$W_{\mathrm{ch}}=\dot{W}\Delta t=\varepsilon \frac{AL^{3}\mu_{\mathrm{air}}}{\tau k}\left[t_{\mathrm{ch}}\left(\upsilon^{2}+\varepsilon \frac{L}{\tau }\frac{\rho_{\mathrm{air}}k}{\mu_{\mathrm{air}}k_{1}}\upsilon^{3}\right)\right].$$

The total amount of CO_2_ collected at time $t_{\mathrm{ch}}$ in the sorbent material of volume V=AL is

(65) $$n_{\mathrm{ch}}={\bar{\beta }}_{\mathrm{ch}}\left[\beta_{0}a_{\mathrm{act}}\right]\left[\rho_{\mathrm{mat}}AL\right]$$

where $\beta_{0}a_{\mathrm{act}}$ is the number of adsorption sites per mass of material, and $\rho_{\mathrm{mat}}AL$ is the mass in the volume AL.

With the reference work

(66) $${\bar{w}}_{0}=\frac{\varepsilon \mu_{\mathrm{air}}L^{2}{\left(\phi +1\right)}^{2}}{\tau k\beta_{0}a_{\mathrm{act}}\rho_{\mathrm{mat}}}\;$$

the pump work per mole of CO_2_ adsorbed assumes the compact form

(67) $$\frac{W_{\mathrm{ch}}}{n_{\mathrm{ch}}}={\bar{w}}_{0}w_{\mathrm{ch}},$$

where $w_{\mathrm{ch}}$ is the dimensionless pump work required for charging the sorbent, which is inversely proportional to the overall charging rate ${\dot{\beta }}_{\mathrm{ch}}=\frac{{\bar{\beta }}_{\mathrm{ch}}}{t_{\mathrm{ch}}}$:

(68) $$w_{\mathrm{ch}}=\frac{1}{{\dot{\beta }}_{\mathrm{ch}}}\left[\frac{1}{\lambda^{2}}+\frac{\gamma }{\lambda^{3}}\right];$$

with the dimensionless inertial parameter

(69) $$\gamma =\varepsilon \left(\phi +1\right)\frac{L}{\tau }\frac{\rho_{\mathrm{air}}k}{\mu_{\mathrm{air}}k_{1}}.$$

Table 3 shows the data for the material considered by Climeworks [36], as well as the resulting values for reference work ${\bar{w}}_{0}$ and inertial parameter γ.

The dimensionless work $w_{\mathrm{ch}}$ depends on the charging rate ${\dot{\beta }}_{\mathrm{ch}}$ and wave parameter λ. Table 4 shows the air usage factor and the dimensionless work for the charging rates of Table 2 (with γ=0.06).

The work required for the reversible separation of CO_2_ from air is in the order of ${\bar{w}}_{\mathrm{rev}}≃20\;\frac{\mathrm{kJ}}{\mathrm{mol}}$ [38]. The work for pumping is consumed by friction in the pores; hence, it must be considered as an irreversible loss [19,38,39]. For the example material, the reference value ${\bar{w}}_{0}=4.28\;\frac{\mathrm{kJ}}{\mathrm{mol}}$ is about one-fifth of the reversible separation work. With the data in Table 4, the actual pumping work $\bar{w}={\bar{w}}_{0}w_{\mathrm{ch}}$ for λ=0.5 is 1.43 times the overall reversible separation work. The behavior is strongly non-linear, with only 16% of the reversible work at $\lambda =t_{\mathrm{ch}}=2$.

Notably, the reference work (66) depends quadratically on sorbent thickness *L*; hence, it can be reduced significantly by using thinner layers of material.

## 5. Optimal System and Process Conditions

The discussion in the previous sections revealed the main parameters and process conditions to explore the fast and efficient adsorption of CO_2_ from air in porous sorbents.

Specifically, one must distinguish between the material, physical, and process parameters, which will be discussed below.

External parameters that cannot be changed and thus will not be discussed further are as follows: environmental temperature *T*, air mole density ${\bar{\rho }}_{\mathrm{air}}$, atmospheric CO_2_ mole fraction $\chi_{\mathrm{atm}}$, air viscosity $\mu_{\mathrm{air}}$, and gas constant $R_{{\mathrm{CO}}_{2}}$.

### 5.1. Material Parameters

One might state the goal of DAC as collecting CO_2_ from the air in compact facilities at high adsorption rates.

Compact facilities demand sorbents with a high density of adsorption sites, that is, large values of $\rho_{\mathrm{mat}}a_{\mathrm{act}}\beta_{0}$ [unit: $\frac{\mathrm{kg}}{m^{3}}\frac{m^{2}}{\mathrm{kg}}\frac{\mathrm{mol}}{m^{2}}=\frac{\mathrm{mol}}{m^{3}}$], which can be separated into the demand for high material mass density $\rho_{\mathrm{mat}}$ and the high mass-specific number of adsorption sites $a_{\mathrm{act}}\beta_{0}$. The latter is measured through the capacity parameter ϕ (12), which compares the number of adsorption sites relative to the number of CO_2_ molecules in air-filled pores,

(70) $$\phi =\frac{a_{\mathrm{act}}\beta_{0}}{v_{\mathrm{acc}}{\bar{\rho }}_{\mathrm{air}}\chi_{\mathrm{atm}}}.$$

A high capacity is desirable for compactness; hence, the sorbent should have a large internal surface area $a_{\mathrm{act}}$ and large site density $\beta_{0}.$

The rate of adsorption is given through the reference time τ that sets the timescale for DAC processes. Revisiting our above line of arguments, this scale is effectively set in the equation for the number density of unoccupied adsorption sites $\hat{\beta }=1-\beta$ when permanently exposed to air with a full CO_2_ load, which in dimensional form is the simple decay equation

(71) $$\frac{d\hat{\beta }}{dt}=-\frac{\hat{\beta }}{\tau }.$$

This definition allows us to estimate the relevant timescale for any adsorption material or model.

For large adsorption rates, the reference time τ should be as small as possible. In our simple yet instructive model, we identified the timescale as (11)

(72) $$\tau =\frac{\sqrt{2\pi }\beta_{0}}{\sqrt{R_{{\mathrm{CO}}_{2}}T}{\bar{\rho }}_{\mathrm{air}}\chi_{\mathrm{atm}}\varpi }.$$

With the site density $\beta_{0}$ being large for high capacity, small τ requires a large adsorption probability factor ϖ. Equivalent statements will be possible for more elaborate adsorption models, which lie outside the scope of this examination.

For typical materials, τ is measured in hours, and a typical charging duration is measured in multiples of τ; hence, the turnover times for adsorption–desorption cycles are rather long. Materials with shorter associated timescales are desperately needed.

### 5.2. Physical Parameters

The actual size of the system is given through the sorbent volume $V_{\mathrm{mat}}=A_{\mathrm{mat}}L$. With the volume flow of air entering as ${\dot{V}}_{\mathrm{air}}=A_{\mathrm{mat}}v$, the volume flow is proportional to the sorbent cross-section $A_{\mathrm{mat}}$, which thus sets the overall size of the system.

The sorbent thickness *L* defines the length scale. In our evaluation, it appears to non-dimensionalize velocity *v* (see next section), and in the reference work (66)

(73) $${\bar{w}}_{0}=\frac{\varepsilon \mu_{\mathrm{air}}L^{2}{\left(\phi +1\right)}^{2}}{\tau k\beta_{0}a_{\mathrm{act}}\rho_{\mathrm{mat}}}.$$

With work ${\bar{w}}_{0}$ proportional to $L^{2}$, a reduction of layer thickness has a strong influence on reducing pump work.

### 5.3. Process Parameters

With the material chosen and system geometry set, the only available process parameters are the dimensionless air velocity υ or, alternatively, the wave parameter $\lambda =\frac{\phi +1}{\upsilon }$, and the charging rate ${\dot{\beta }}_{\mathrm{ch}}$ or, alternatively, the charging duration $t_{\mathrm{ch}}$.

As our discussion of charging processes has revealed, for efficient operation, these parameters are not independent. Specifically, when the wave parameter λ is chosen, a charging time not too different from $t_{\mathrm{ch}}=\lambda$ yields good use of the material; see Figure 6 and Table 2.

With this, only the wave parameter λ remains to be chosen. With decreasing λ, both, the charging rate ${\dot{\beta }}_{\mathrm{ch}}\left(\lambda \right)$ and the pump work $w_{\mathrm{ch}}\left(\lambda \right)$ are increasing; see Table 2 and Table 4, hence, there is no unique value of λ obtainable from an optimization. One has to choose an appropriate value to strike a balance between the contradictory demands of the large charging rate and small pump work.

While we refrained from a detailed discussion of the desorption part of the overall DAC process, a short comment is in order here. Desorption is a thermal process where the sorbent is heated to a suitable temperature (of the order of 100 °C). Heating and subsequent cooling of the sorbent is an inherently irreversible process, and the best use of the supplied heat will be made when the accumulation is relatively high.

With work increasing quadratically for small λ, the adsorption rate decreasing with large λ, and a desire for relatively large accumulation, we believe that the target value should lie in the range of

(74) λ=2⋯6,

for which the charging rate assumes values in the range $\left[0.35\;\cdots \;0.15\right]$ and the accumulation is in the range of 70–90%. For the sample material and process of Ref. [36], we find λ=5.75, which is at the upper end of our recommendation.

We emphasize that for small layer thickness *L*, the reference work ${\bar{w}}_{0}$ will be small as well; hence, the wave parameter can be smaller for large charging rates without demanding too much work.

Table 5 summarizes the recommendations in compact form.

### 5.4. Physical Size

To close our arguments, we have a brief look at the required size for large-scale DAC facilities. Aiming for a real global impact, we consider CO_2_ removal of $1\frac{t}{\mathrm{year}}$ per person, which is $8\frac{\mathrm{Gt}}{\mathrm{year}}≃10^{6}\frac{t}{h}$. Assuming the capacity of a single plant similar in size to CarbonEngineering’s proposal [17,19] at $100\frac{t}{h}$, this requires 10,000 plants (which is one plant per 800,000 people).

We ask for the mass of sorbent $m_{\mathrm{mat}}$ required for one of these plants. The dimensional overall removal rate is

(75) $${\dot{R}}_{{\mathrm{CO}}_{2}}=\frac{5}{6}m_{\mathrm{mat}}M_{{\mathrm{CO}}_{2}}a_{\mathrm{act}}\frac{\beta_{0}}{\tau }{\dot{\beta }}_{\mathrm{ch}}$$

where the factor $\frac{5}{6}$ results from the assumption that the time for desorption is $\frac{1}{5}$ of the time required for adsorption, so that five of six unit times are used for charging.

We evaluate this for the sample material from the Climeworks patent [36], as listed in Table 1 and Table 3. Choosing λ=4 with ${\dot{\beta }}_{\mathrm{ch}}=0.2$, we find the required mass of the sorbent as

(76) $$m_{\mathrm{mat}}=\frac{6}{5}\frac{{\dot{R}}_{{\mathrm{CO}}_{2}}\tau }{M_{{\mathrm{CO}}_{2}}a_{\mathrm{act}}\beta_{0}{\dot{\beta }}_{\mathrm{ch}}}=27,300\;t,$$

which fills the volume

(77) $$V_{\mathrm{mat}}=\frac{m_{\mathrm{mat}}}{\rho_{\mathrm{mat}}}=49,000\;m^{3},$$

corresponding to a cube with an edge length of 36.6m.

With a layer thickness of L=0.04m, the cross-section for the inflow is

(78) $$A_{\mathrm{mat}}=\frac{V_{\mathrm{mat}}}{L}=1.23\times 10^{6}\;m^{2}.$$

Obviously, all numbers are scaled with the characteristic time τ; hence, a decrease of τ, that is, using materials with a faster intake of CO_2_ is essential.

Using the above numbers, we find a sorbent volume of $250\;m^{3}$ for Climeworks’ 4000 t/year Orca plant, and $2250\;m^{3}$ for their 36,000 t/year Mammoth plant.

These estimates do not account for the adsorption of moisture from the air, which reduces the number of sites available for CO_2_ molecules.

The data used here, as extracted from Ref. [36], are probably outdated, but we were not able to find data for newer materials for further evaluation. Certainly, the above numbers provide some idea of the significant physical size of the facilities that direct air capture of CO_2_ demands.

## 6. Conclusions

A reduction in CO_2_ emissions as well as direct air capture (DAC) of CO_2_ are important tasks for humankind to reduce climate change. Above, we have studied the adsorption step in DAC systems by means of a simple but realistic model, where non-dimensionalization and mathematical evaluation identified the key parameters for these processes: the sorbent material is described through the adsorption timescale τ and its adsorption capacity ϕ, and through layer thickness *L*, while the running of the process depends on dimensionless air velocity υ and charging duration $t_{\mathrm{ch}}$.

Since the charging processes are governed only by the wave parameter $\lambda =\frac{\phi +1}{\upsilon }$, process control requires only to chose a suitable value for λ as well as the corresponding charging duration $t_{\mathrm{ch}}$. Our model allows a systematic and quick evaluation of different choices that help with decision-making. While larger velocities, hence smaller values of λ, lead to faster charging rates, they imply considerably larger work demand, and as such, one will have to strike a compromise between the CO_2_ collection rate and work requirements. Our evaluation leads to the recommendation that values in the range of λ=2…6 with $t_{\mathrm{ch}}=\lambda$ should be considered for sorbent charging.

While the adsorption model used is relatively simple, it accounts for the proper physics of the problem. That is, more refined models will behave quite similarly, and, in particular, they will describe wave-like transport governed by a corresponding wave parameter (see Appendix A). Hence, the discussion of DAC performance measures and behavior will be rather similar to the above.

We hope that the instructive model and its evaluation will be helpful for further research into DAC adsorption processes, as well as being educational for those wishing to understand such processes better.

## Figures and Tables

**Table 1:** Data for the material described in [36].

Parameter	Value	
${\bar{\rho }}_{\mathrm{air}}$	$42.5\;\frac{\mathrm{mol}}{m^{3}}$	standard air
$\chi_{\mathrm{atm}}$	$4.2\times 10^{-4}$	mole fraction of CO_2_ in atmosphere
$\beta_{0}$	$4.6\times 10^{-6}\;\frac{\mathrm{mol}}{m^{2}}$	adsorption site density
$a_{\mathrm{act}}$	$3.04\times 10^{5}\;\frac{m^{2}}{\mathrm{kg}}$	active area per mass
$v_{\mathrm{acc}}=\frac{1}{\rho_{\mathrm{mat}}}$	$1.79\times 10^{-3}\;\frac{m^{3}}{\mathrm{kg}}$	accessible volume per mass
*L*	0.04m	thickness of slab
$A_{\mathrm{tot}}$	$1.25\times 10^{-3}\;m^{2}$	cross section of slab
*Q*	$3.33\times 10^{-5}\;\frac{m^{3}}{s}$	air mass flow rate
$v=\frac{Q}{A_{\mathrm{tot}}}$	$2.67\times 10^{-2}\;\frac{m}{s}$	flow speed
D	$1.6\times 10^{-5}\;\frac{m^{2}}{s}$	CO_2_ in air diffusion coefficient

**Table 2:** Charging conditions for $t_{\mathrm{ch}}=\lambda$ from numerical solution; values in brackets are for the wave solution.

$\lambda =t_{\mathrm{ch}}$	0.5	1	2	4	6	8	10	12
${\bar{\beta }}_{\mathrm{ch}}\left(t_{\mathrm{ch}}\right)$	$\begin{array}{c} 0.335\\ \left(0.562\right) \end{array}$	$\begin{array}{c} 0.509\\ \left(0.620\right) \end{array}$	$\begin{array}{c} 0.688\\ \left(0.717\right) \end{array}$	$\begin{array}{c} 0.829\\ \left(0.831\right) \end{array}$	$\begin{array}{c} 0.886\\ \left(0.885\right) \end{array}$	$\begin{array}{c} 0.913\\ \left(0.913\right) \end{array}$	$\begin{array}{c} 0.931\\ \left(0.931\right) \end{array}$	$\begin{array}{c} 0.942\\ \left(0.942\right) \end{array}$
${\dot{\beta }}_{\mathrm{ch}}=\frac{{\bar{\beta }}_{\mathrm{ch}}\left(t_{\mathrm{ch}}\right)}{t_{\mathrm{ch}}}$	$\begin{array}{c} 0.67\\ \left(1.12\right) \end{array}$	$\begin{array}{c} 0.509\\ \left(0.620\right) \end{array}$	$\begin{array}{c} 0.344\\ \left(0.358\right) \end{array}$	$\begin{array}{c} 0.207\\ \left(0.208\right) \end{array}$	$\begin{array}{c} 0.148\\ \left(0.147\right) \end{array}$	$\begin{array}{c} 0.114\\ \left(0.114\right) \end{array}$	$\begin{array}{c} 0.093\\ \left(0.093\right) \end{array}$	$\begin{array}{c} 0.079\\ \left(0.079\right) \end{array}$

**Table 3:** Additional data for the material descibed in [36].

Parameter	Value	
$\rho_{\mathrm{air}}$	$1.3\;\frac{\mathrm{kg}}{m^{3}}$	mass density of air
$\mu_{\mathrm{air}}$	$1.81\times 10^{-5}\;\frac{\mathrm{kg}}{ms}$	viscosity of air
$\rho_{\mathrm{mat}}$	$558\;\frac{\mathrm{kg}}{m^{3}}$	mass density of sorbent
ε	0.43	void fraction
$d_{p}$	$6.2\times 10^{-4}\;m$	particle diameter
*k*	$6.3\times 10^{-10}\;m^{2}$	permeability
$k_{1}$	$4.9\times 10^{-5}\;m$	inertial permeability
γ	0.06	inertial parameter
${\bar{w}}_{0}$	$4.28\;\frac{\mathrm{kJ}}{\mathrm{mol}}$	reference work for pump

**Table 4:** Air usage factor Π and dimensionless work $w_{\mathrm{ch}}$ for charging duration $t_{\mathrm{ch}}=\lambda$ (from numerical solution).

$\lambda =t_{\mathrm{ch}}$	0.5	1	2	4	6	8	10	12
Π	0.335	0.509	0.688	0.829	0.886	0.913	0.931	0.942
$w_{\mathrm{ch}}$	6.69	2.08	0.749	0.306	0.190	0.138	0.108	0.088

**Table 5:** Material, physical, and process parameters, and their required range of values. See text for details.

Name	Symbol	Unit	Requirement
sorbent density	$\rho_{\mathrm{mat}}$	$\frac{\mathrm{kg}}{m^{3}}$	“large”
sorbent active area	$a_{\mathrm{act}}$	$\frac{m^{2}}{\mathrm{kg}}$	“large”
sorbent saturation capacity	$\beta_{0}$	$\frac{\mathrm{mol}}{m^{2}}$	“large”
capacity factor	ϕ	1	≫1
reference time	τ	s	min, not h
probability factor	ϖ	1	0≪ϖ≤1
sorbent thickness	*L*	m	as small as possible
reference work	${\bar{w}}_{0}$	$\frac{\mathrm{kJ}}{\mathrm{mol}}$	$\ll {\bar{w}}_{\mathrm{rev}}=20\frac{\mathrm{kJ}}{\mathrm{mol}}$
pump work	$w_{\mathrm{ch}}$	1	“small”
charging duration	$t_{\mathrm{ch}}$	1	=λ, “small”
charging rate	${\dot{\beta }}_{\mathrm{ch}}$	1	“large”
air velocity	$\upsilon =\frac{\tau }{L}v$	1	$=\frac{\phi +1}{\lambda }$
wave parameter	λ	1	2⋯6 (balanced)

## Data Availability

The original contributions presented in the study are included in the article, further inquiries can be directed to the corresponding author.

## Appendix A. Adsorption Rate and the Second Law

### Appendix A.1. Transport Equations

We ask for the formulation of the second law of thermodynamics for the transport model (15) and (16). To this end, we consider the adsorption process akin to a chemical reaction where CO_2_ (χ) and free adsorption sites ($\hat{\beta }$) “react” to form occupied adsorption sites (β). To proceed, we write the dimensionless reaction diffusion system as

(A1) $$\begin{array}{ccc} \frac{\partial \phi \beta }{\partial t} & = & \Lambda \end{array}$$

(A2) $$\begin{array}{ccc} \frac{\partial \phi \hat{\beta }}{\partial t} & = & -\Lambda \end{array}$$

(A3) $$\begin{array}{ccc} \frac{\partial \chi }{\partial t}+\upsilon \frac{\partial \chi }{\partial x}-D\frac{\partial^{2}\chi }{\partial x^{2}} & = & -\Lambda \end{array}$$

where $\Lambda \left(x,t\right)$ is the adsorption rate. In this dimensionless formulation the factor ϕ accounts for adjusting units between the variables, which in dimensional form are surface densities β, $\hat{\beta }$, and mole fraction χ. The number of adsorption sites are constant, hence

(A4) $$\beta +\hat{\beta }=1.$$

### Appendix A.2. Linear Irreversible Thermodynamics

We proceed with the standard methods from nonequilibrium thermodynamics for the modeling of reactions, see, e.g., [40], which here simplify significantly since we consider only fixed temperatures and pressures. Then, it suffices to consider the dimensionless entropy density as

(A5) $$\eta =\chi \left(s_{\chi }-\ln\chi \right)+\phi \hat{\beta }\left(s_{\hat{\beta }}-\ln\hat{\beta }\right)+\phi \beta \left(s_{\beta }-\ln\beta \right)$$

where the $s_{\alpha }$ are entropy constants. Taking the time derivative of η and replacing the time derivatives of the variables β, $\hat{\beta }$, χ by means of the transport equations yields after some standard manipulation of the second law of thermodynamics in the form [39,40]

(A6) $$\frac{\partial \eta }{\partial t}+\frac{\partial \varphi }{\partial x}=\sigma$$

with the entropy flux

(A7) $$\varphi =\upsilon \chi \left(s_{\chi }-\ln\chi \right)-\left(D\left(s_{\chi }-\ln\chi -1\right)\frac{\partial \chi }{\partial x}\right)$$

and the entropy production rate

(A8) $$\sigma =\left[1+s_{\beta }-s_{\chi }-s_{\hat{\beta }}+\ln\frac{\chi \hat{\beta }}{\beta }\right]\Lambda +\frac{D}{\chi }\frac{\partial \chi }{\partial x}\frac{\partial \chi }{\partial x}.$$

The entropy production consists of two contributions, which must be independently positive. The second term describes the entropy generation due to the diffusion of CO_2_ in air, and is positive for the positive diffusion coefficient *D*. The first term describes entropy generation in the adsorption process and will now be used to model the adsorption rate Λ.

To proceed, we define the equilibrium constant k by writing

(A9) $$\ln k=s_{\chi }+s_{\hat{\beta }}-s_{\beta }-1,$$

which gives the adsorption contribution to entropy generation as

(A10) $$\sigma_{\mathrm{ad}}=\left[\ln\chi \hat{\beta }-\ln k\beta \right]\Lambda ⩾0.$$

Following the principles of classical linear irreversible thermodynamics [39,40], we interpret this expression as the product of the thermodynamic force $F=\left[\ln\chi \hat{\beta }-\ln k\beta \right]$ and the thermodynamic flux J=Λ. For close to equilibrium processes, one tends to express the flux in a linear relation—a phenomenological equation—of the form J=αF with phenomenological coefficient α, but for chemical reactions and the adsorption process studied here, these are not useful. Instead, as is often done in reaction kinetics [40], we consider an Arrhenius-type ansatz by taking the exponentials of the two competing terms in the force as

(A11) $$\Lambda =\phi \left[\exp\left[\ln\chi \hat{\beta }\right]-\exp\left[\ln k\beta \right]\right]=\phi \left[\chi \hat{\beta }-k\beta \right],$$

where the factor ϕ is chosen for agreement with the proposed model equations. The adsorption model studied in the bulk of this paper arises in the limit k→0, which implies that the spontaneous detachment of CO_2_ from an occupied site is effectively impossible. This is similar to the analysis of combustion processes, where only forward reactions are considered.

The chemical constant depends on temperature, $k\left(T\right)$, and increases with temperature so that desorption occurs at larger temperatures.

### Appendix A.3. Langmuir Isotherm

In equilibrium, the reaction rate must vanish, $\Lambda_{eq}=0$, which gives the Langmuir isotherm as, with $\beta +\hat{\beta }=1$,

(A12) $$\beta_{eq}=\frac{\chi }{k+\chi }.$$

In equilibrium with the atmosphere, in our dimensionless formulation, we have $\chi_{atm}=1$, hence

$$\beta_{eq}=\frac{1}{1+k},\;\;\;{\hat{\beta }}_{eq}=1-\beta_{eq}=\frac{k}{1+k}.$$

For the case k→0, this reduces to the case studied in the main paper, where $\beta_{eq}=1$, ${\hat{\beta }}_{eq}=0$.

### Appendix A.4. Wave Solution

Also, with k≠0, the adsorption process is wave-like. For a wave connecting the equilibrium with air in the wake at $\chi_{-\infty }=1$ and the post-desorption equilibrium state $\chi_{\infty }$, the wave solution is obtained from a straightforward calculation as

(A13) $$\begin{array}{ccc} \chi \left(x,t\right) & = & \frac{1+\chi_{\infty }}{2}+\frac{1-\chi_{\infty }}{2}\tanh\left[-\frac{1-\chi_{\infty }}{2}\left(\lambda x-t-\xi_{0}\right)\right] \end{array}$$

(A14) $$\begin{array}{ccc} \hat{\beta }\left(x,t\right) & = & \frac{k}{1+k}\left[1+\frac{1-\chi \left(x,t\right)}{k+\chi_{\infty }}\right] \end{array}$$

(A15) $$\begin{array}{ccc} \beta \left(x,t\right) & = & \frac{1}{1+k}\left[1-\frac{k\left(1-\chi \left(x,t\right)\right)}{k+\chi_{\infty }}\right] \end{array}$$

with the wave parameter

(A16) $$\lambda =\frac{1}{\alpha \upsilon }=\frac{\frac{\phi +1}{\upsilon }}{1+\frac{\chi_{\infty }}{k}}+\frac{k^{2}\phi +\chi_{\infty }}{\upsilon \left(k+\chi_{\infty }\right)}.$$

We recognize the coefficient λ as the inverse wave speed, while the factor $\left(1-\chi_{\infty }\right)\lambda$ determines the width of the wave, which is wider for smaller values, i.e., larger $\chi_{\infty }$.

The two equilibrium states are, in the wake of the wave: ξ→−∞ with $\tanh\left(\infty \right)=1$

(A17) $$\chi_{-\infty }=1,\;\;\;{\hat{\beta }}_{-\infty }=\frac{k}{1+k},\;\;\;\beta_{-\infty }=\frac{1}{1+k};$$

and in front of the wave: ξ→∞ with $\tanh\left(-\infty \right)=-1$

(A18) $$\chi_{\infty },\;\;\;{\hat{\beta }}_{\infty }=\frac{k}{k+\chi_{\infty }},\;\;\;\beta_{\infty }=\frac{\chi_{\infty }}{k+\chi_{\infty }}.$$

The waves discussed in the bulk of the paper result in the limit k→0, which also requires $\chi_{\infty }=0$, so that in the limit k→0, we find ${\hat{\beta }}_{\infty }=1,\beta_{\infty }=0$.
